# A Statewide Nested Case–Control Study of Preterm Birth and Air Pollution by Source and Composition: California, 2001–2008

**DOI:** 10.1289/ehp.1510133

**Published:** 2016-02-19

**Authors:** Olivier Laurent, Jianlin Hu, Lianfa Li, Michael J. Kleeman, Scott M. Bartell, Myles Cockburn, Loraine Escobedo, Jun Wu

**Affiliations:** 1 Program in Public Health, University of California, Irvine, Irvine, California, USA; 2 Department of Civil and Environmental Engineering, University of California, Davis, Davis, California, USA; 3 School of Environmental Science and Engineering, Nanjing University of Information Science and Technology, Nanjing, Jiangsu, China; 4 Department of Statistics, University of California, Irvine, Irvine, California, USA; 5 Keck School of Medicine, University of Southern California, Los Angeles, California, USA

## Abstract

**Background:**

Preterm birth (PTB) has been associated with exposure to air pollution, but it is unclear whether effects might vary among air pollution sources and components.

**Objectives:**

We studied the relationships between PTB and exposure to different components of air pollution, including gases and particulate matter (PM) by size fraction, chemical composition, and sources.

**Methods:**

Fine and ultrafine PM (respectively, PM2.5 and PM0.1) by source and composition were modeled across California over 2000–2008. Measured PM2.5, nitrogen dioxide, and ozone concentrations were spatially interpolated using empirical Bayesian kriging. Primary traffic emissions at fine scale were modeled using CALINE4 and traffic indices. Data on maternal characteristics, pregnancies, and birth outcomes were obtained from birth certificates. Associations between PTB (n = 442,314) and air pollution exposures defined according to the maternal residence at birth were examined using a nested matched case–control approach. Analyses were adjusted for maternal age, race/ethnicity, education and neighborhood income.

**Results:**

Adjusted odds ratios for PTB in association with interquartile range (IQR) increases in average exposure during pregnancy were 1.133 (95% CI: 1.118, 1.148) for total PM2.5, 1.096 (95% CI: 1.085, 1.108) for ozone, and 1.079 (95% CI: 1.065, 1.093) for nitrogen dioxide. For primary PM, the strongest associations per IQR by source were estimated for onroad gasoline (9–11% increase), followed by onroad diesel (6–8%) and commercial meat cooking (4–7%). For PM2.5 composition, the strongest positive associations per IQR were estimated for nitrate, ammonium, and secondary organic aerosols (11–14%), followed by elemental and organic carbon (2–4%). Associations with local traffic emissions were positive only when analyses were restricted to births with residences geocoded at the tax parcel level.

**Conclusions:**

In our statewide nested case–control study population, exposures to both primary and secondary pollutants were associated with an increase in PTB.

**Citation:**

Laurent O, Hu J, Li L, Kleeman MJ, Bartell SM, Cockburn M, Escobedo L, Wu J. 2016. A statewide nested case–control study of preterm birth and air pollution by source and composition: California, 2001–2008. Environ Health Perspect 124:1479–1486; http://dx.doi.org/10.1289/ehp.1510133

## Introduction

Preterm birth (PTB) is defined as birth before 37 completed weeks of gestation (March of Dimes, PMNCH, Save the Children, and WHO 2012). PTB is a major cause for infant death and morbidity, and has also been associated with adverse effects later in life including impaired vision, hearing, and cognitive function, decreased motor function, and behavioral disorders (Saigal and Doyle 2008). Air pollution has been hypothesized to increase the risk of PTB, notably by increasing systemic oxidative stress and inflammation (Vadillo-Ortega et al. 2014), impairing placentation (van den Hooven et al. 2009), causing endocrine disruption (e.g., disturbing the pituitary–adrenocortico–placental system), and increasing maternal susceptibility to infections (Slama et al. 2008). A growing number of studies have reported positive associations between exposure of pregnant women to air pollution and PTB (Kloog et al. 2012; Olsson et al. 2013; Pereira et al. 2014; Stieb et al. 2012; Wilhelm et al. 2011), although results vary widely among studies: Positive associations have been reported between particulate matter (PM) and PTB in some studies (e.g., Kloog et al. 2012; Pereira et al. 2014; Stieb et al. 2012; Wu et al. 2009b), whereas inverse associations have been reported in others (e.g., Trasande et al. 2013; Wilhelm et al. 2011). Beyond the possible influences of methodological differences and of varying population susceptibilities between study settings, such discrepancies might also be attributable to differences in PM composition across settings. Potential effects of PM on PTB might be mediated by core chemical components of PM (e.g., elemental carbon, nitrates) or by organic compounds [e.g. quinones, polycyclic aromatic hydrocarbons (PAHs)] or metals adsorbed onto the particle surface (Schlesinger et al. 2006). PM composition varies highly across seasons and settings (Bell et al. 2007). To our knowledge, only two U.S. studies have examined the association between PM composition and PTB. In Los Angeles County, California, organic carbon (OC), elemental carbon (EC), and ammonium nitrate in fine PM (PM_2.5_; ≤ 2.5 μm in aerodynamic diameter) were positively associated with PTB, despite an inverse association with total PM_2.5_ mass (Wilhelm et al. 2011). In Atlanta, Georgia, sulfate and water-soluble metals in PM_2.5_ were positively associated with PTB despite a lack of association with total PM_2.5_ mass (Darrow et al. 2009).

The composition of air pollution, and any related health risk that depends on composition, is influenced by the nature of contributing air pollution sources. Identifying the sources most likely to cause PTB is not only a question of scientific interest but also of policy relevance. A large number of studies have examined the relationships between PTB and traffic-related pollutants or proximity to traffic sources. They generally reported positive associations (Généreux et al. 2008; Miranda et al. 2013; Wu et al. 2009b; Yorifuji et al. 2011), with some exceptions (Brauer et al. 2008; Malmqvist et al. 2011). Only a few studies examined PTB in relation to geographical proximity to other sources [oil refineries (Yang et al. 2004), cement plants (Yang et al. 2003), or gasoline stations (Huppé et al. 2013)] or to exposure to PM from specific sources [e.g., open-hearth steel mill (Parker et al. 2008), coal (Mohorovic 2004), diesel (Wilhelm et al. 2011), or biomass burning (Wilhelm et al. 2011; Wylie et al. 2014)]. Only one study examined the association between PTB and PM from several sources within an integrated framework (Wilhelm et al. 2011), which is needed to allow for a rigorous comparison of source influence on PTB and identification of the most harmful sources.

Finally, to the best of our knowledge, the relationship between PTB and ultrafine PM (PM_0.1_; ≤ 0.1 μm in aerodynamic diameter) has never been studied. Important concerns exist regarding the toxicity of particles in the PM_0.1_ size fraction due to their larger number concentrations and surface-to-volume ratios relative to fine or coarse PM (Knol et al. 2009), yielding a higher total surface area available for adsorption of toxic chemicals such as metals or PAHs. Ultrafine PM also have higher potential than fine or coarse PM for translocation into organs other than the lung and even into cells (Schlesinger et al. 2006).

In this work we aimed to study the relationships between air pollution and preterm births that occurred during 2001–2008 throughout the state of California. We extended previous research on this topic by using spatiotemporal chemical transport modeling of particles by source and composition and by studying PM_0.1_ exposure. We also employed more commonly used air pollution metrics such as interpolated measurement data, predictions from a line source dispersion model, traffic density, and proximity to roads. These methods enabled the comparison of the estimated effects of different components and sources of air pollution within a consistent framework, in an attempt to identify those most strongly associated with PTB.

## Methods

### Air Pollution Metrics

***Empirical Bayesian kriging of monitoring station measurements.*** Measurements from monitoring stations throughout the state for years 2000–2008 were obtained from the California Air Resources Board (http://www.arb.ca.gov/) for total PM_2.5_, nitrogen dioxide (NO_2_), and ozone (O_3_). Only results from filter-based measurements, generally conducted every 3 or 6 days, were included for PM_2.5_. Hourly gaseous pollutant measurements were converted to daily means using a criterion of 75% data completeness at a 24-hr basis. Only data for the 1000- to 1800-hours time windows were used to calculate daily means for O_3_. Monthly averages for pollutants were then calculated for stations with > 75% days of valid data in a month. These monthly averaged concentrations were spatially interpolated between stations using an empirical Bayesian kriging (EBK) model (Pilz and Spöck 2007) implemented in ArcGIS 10.1 (ESRI, Redlands, CA). Because of the high computational cost, we applied this approach only to monthly averaged concentrations. The number of available monitors per month varied during the study period (ranges, 75–98 for PM_2.5_, 151–182 for O_3_, and 94–109 for NO_2_). Pollutant surface predictions were generated for 200 m × 200 m grids (Wu et al. 2016). Leave-one-out cross-validation was conducted for model evaluation. A single sample of monthly averaged measurement data (at one station) was selected as the test sample while other samples (at the other stations) were used to train the EBK models. This process was repeated so that every monthly sample was estimated independently as the validation data. The resulting *R* ^2^ and root mean square error (RMSE) estimated were *R* ^2^ = 0.65 and RMSE = 3.65 μg/m^3^ for PM_2.5_; *R* ^2^ = 0.74 and RMSE = 6.08 ppb for NO_2_; *R* ^2^ = 0.72; and RMSE = 5.81 ppb for O_3_ (Wu et al. 2016).

***Chemical transport modeling.*** The daily mass concentration of primary PM (PM emitted directly into the atmosphere) and of secondary PM (formed in the atmosphere from gas-phase precursors) was estimated at 4-km spatial resolution across two domains covering 92% of the California population for the period of 2000–2008, using the University of California-Davis/California Institute of Technology (UCD/CIT) chemical transport model (Hu et al. 2015). The UCD/CIT model includes a complete description of atmospheric transport, deposition, chemical reaction, and gas-particle transfer (Hu et al. 2015). This model provided mass concentration estimates for primary PM total mass and for several chemical species in PM [OC, EC, nitrates, sulfates, ammonium and secondary organic aerosols (SOA)].

In addition, the University of California Davis/CIT_Primary (UCD_P) chemical transport model was used across the same geographical domain for the period of 2000–2006 to predict the daily mass concentrations for further chemical species and for the total mass of primary PM broken down by source (Hu et al. 2014a, 2014b). The model simulated daily primary PM mass concentrations, also at a 4 km × 4 km grid resolution, from ~ 900 sources. Composition profiles were applied combined with the primary PM mass concentration predictions from the UCD_P model to estimate the concentrations of chemical species in primary PM. The mass, source, and composition of size-resolved PM were simulated by model calculations. We decided *a priori* to include in our analyses UCD_P estimates of sources and components of primary PM for which previously published detailed validation results were available. Sets of validation results spanned multiple years between 2000 and 2007 (Hu et al. 2014a, 2014b). Previous analyses were conducted to directly evaluate the accuracy of simulated source contributions using the UCD models. These tests included comparison to receptor-oriented source apportionment studies at multiple locations throughout California (Hu et al. 2014b). Onroad gasoline, diesel, commercial meat cooking, and wood burning passed two complex source constraint checks based on *a*) comparison with tracer-based source-apportionment studies and *b*) model performance for individual species defined by four criteria (see “Description of the source constraint checks performed to directly evaluate the accuracy of simulated source contributions using the UCD_P model” in the Supplemental Material). Further analysis compared predicted components of PM mass including elemental carbon and various trace metals to measurements at several locations throughout California. We decided *a priori* to select for the present epidemiological analyses only primary PM components for which correlations between monthly averaged predictions and measured values were > 0.8 in the PM_2.5_ size fraction (because this fraction has the greatest number of available measurements). Nine species of PM (potassium, chromium, iron, titanium, magnesium, strontium, arsenic, calcium, and zinc) matched this criterion (Hu et al. 2014a).

***CALINE4 dispersion modeling for road sources.*** A modified version of CAlifornia LINE Source Dispersion Model Version 4 (CALINE4) (Benson 1989; Wu et al. 2009a) was used to predict ambient concentrations from local traffic emissions of carbon monoxide (CO), nitrogen oxides (NO_x_), and ultrafine particle number (UFP) up to 3 km from maternal residences (Yuan et al. 2011). Model inputs included roadway geometry and traffic counts, emission factors, and meteorological parameters (wind direction, wind speed, temperature stability class, and mixing heights). CALINE4 predictions were not conducted for 5% of births, for which no traffic count data were recorded within 3 km of the maternal residences. CALINE4 predictions in this study did not incorporate background levels of pollutants, and thus solely represent the contribution from local traffic emissions (Wu et al. 2016). We compared the CALINE4-modeled daily UFP number concentrations with particle number concentrations measured using the Condensation Particle Counter (model 3785; TSI, Inc., Shoreview, MN) at four monitoring sites located in southern California from a separate study (Delfino et al. 2010). The measurements contained 86–92 days of data at each site, with a total of 357 days of measurements from all the sites. The overall correlation between the modeled and the measured concentrations of particle numbers was 0.75. More details about the model evaluation can be found elsewhere (Wu et al. 2016).

***Traffic and roadways.*** Traffic densities within circular buffers of different sizes centered on maternal homes were calculated based on 2002 annual average daily traffic counts (AADT) data from the California Department of Transportation (CALTRANS 2012). To estimate traffic density, AADT on each road segment was weighted by the length of this same road segment within the buffer. These traffic densities for 2002 were then scaled to other years based on temporal trends in total vehicle miles traveled (from 2000 through 2008) in California (CALTRANS 2013).

U.S. major roads data based on TeleAtlas streets (ESRI 2010) were used to calculate the distance from each maternal home to the nearest major road [defined by categories of functional road classes (FRC) A0–A5].

### Study Population

Birth certificate records for all births occurring from 1 January 2001 through 31 December 2008 in California (*n* = 4,385,997) were obtained from the California Department of Public Health. Maternal addresses of residence recorded on birth certificates were geocoded using the University of Southern California GIS Research Laboratory geocoding engine (Goldberg et al. 2008), which geocoded maternal residences at the centroid of tax parcels whenever feasible. In total, we had 54.02% of addresses geocoded within a parcel. Further, we had 37.23% of all births that were geocoded within 50 m of a parcel. In addition, 8.55% of addresses were in California but not within or close to a parcel, and they were geocoded to the centroid of the ZIP code or city whenever feasible. In total, 1,361 births had no usable coordinates at all, and 7,512 infants were born to women residing outside of California. After excluding these births and those who had state file number information missing (*n* = 8,119, partially overlapping with births that lacked usable coordinates or occurred outside of California), we obtained birth certificate data for 4,370,371 pregnancies.

Infants with recorded birth defects or unknown birth defects status (*n* = 18,811 and *n* = 675, respectively) were excluded. The time of conception and resulting gestational age (in days) were estimated based on the first day of the last menstrual period reported by mothers. We excluded birth with missing information for gestational age (*n* = 196,247), estimated gestational age < 121 or > 319 days (*n* = 2,051 and *n* = 41,017, respectively), implausible combinations of birth weight and gestational age (*n* = 17,026) (Alexander et al. 1996), or infants born to mothers > 60 years of age (*n* = 43). Infants conceived > 19 weeks before the start (1 January 2001), or < 43 weeks before the end (31 December 2008) of the study (*n* = 389,611 and *n* = 38,598, respectively) were further excluded to avoid fixed cohort bias (Strand et al. 2011). Several exclusion criteria overlapped for certain births, leaving 3,870,696 births from the source population eligible for the study. All PTB cases (infants born < 37 gestational weeks) from the source population that met the eligibility criteria (*n* = 442,314) were included in the present study. For each PTB case, two controls (infants born ≥ 37 gestational weeks) matched on the calendar year of conception (determined from the estimated date of conception, as explained above) were randomly selected from the source population without replacement. The same approach was employed for sensitivity analyses of moderately preterm births (MPTB cases, born < 35 weeks, *n* = 158,645 and controls born at ≥ 35 weeks) and very preterm births (VPTB cases, born < 30 weeks, *n* = 29,510 and controls born ≥ 30 weeks).

### Statistical Analyses

We used a nested matched case–control approach to analyze the association between each air pollutant and PTB (or MPTB or VPTB) (Huynh et al. 2006; Wilhelm et al. 2011). This approach was used instead of a full cohort analysis because of the computational difficulties in fitting models with about 4,000,000 observations. Because controls were randomly selected from a source cohort population basis, this design is free from selection biases often encountered in classical case–control studies and produces results comparable with those of a cohort analysis with little loss in efficiency (Kass and Gold 2007). Because by definition cases are born preterm and controls experience a longer gestation time, cases and controls were exposed to air pollution during different periods of gestation. To allow for a valid comparison of exposures between cases and controls, for each control we truncated exposure estimates at the gestational age reached by the PTB (or MPTB or VPTB) case to which it had been matched. To account for this risk set design, conditional logistic regression was employed for the analysis of the association between air pollution and preterm birth, using the “survival” package of the R environment, version 3.0.1. (R Core Team 2013). Robust standard errors were estimated (Lee et al. 2013). Inferences were based on statistical significance at the 5% level.

For pollutant measurements interpolated by EBK (total PM_2.5_, O_3_, and NO_2_), for UCD_P, UCD/CIT, and CALINE4 predictions, we conducted analyses for “average pregnancy exposures” (which for controls was actually truncated at the gestational age in days reached by cases to which they were matched). We conducted analyses according to exposure categories for pollutant concentrations, defined as quartiles of the exposure metric distribution in the case–control set. We also introduced air pollution metrics as linear terms in the models and then report odds ratios (ORs) for PTB for an interquartile range (IQR) in air pollution metrics. IQRs were derived separately for each model, so that IQRs for the same pollutant may vary between the main analysis and sensitivity analyses. To allow for the comparison of associations between PTB and traffic density across buffers of different sizes, we scaled risk estimates to an increase of 10,000 vehicles per day per meter for this exposure metric. Distance to roadway was analyzed using dichotomous indicators for living or not living within certain distances from roads.

Risk factors for PTB other than air pollution were identified from the literature, and a causal diagram was drawn (see Figure S1) to identify the minimal set of potential confounders to adjust for (Greenland et al. 1999). In our primary analyses we adjusted for educational level (in categories defined as follows: ≤ 8th grade, 9th grade to high school, and college education), maternal race/ethnicity (in mutually exclusive categories as follows: African American, Asian, Hispanic regardless of race, non-Hispanic white, and others including Hawaiian/Pacific Islanders, American Indian/Alaskan native and mothers with multiple race/ethnicities specified), maternal age, and median household income by census block group (U.S. Census Bureau 2004), using quadratic polynomial functions. However, we acknowledge uncertainties in our causal diagram; incorrect assessment of the causal relationships could affect selection of the minimal set of potential confounders for adjustment. We therefore examined the effects of further adjustment for body mass index (BMI) at the beginning of pregnancy or for smoking during pregnancy in addition to the covariates included in the primary model, in the subset of infants born in 2007 and 2008, because these variables were not recorded on birth certificates in the previous years.

The use of two-pollutant models was explored for measured ambient concentrations interpolated with EBK. Last, for traffic indicators at fine geographic scale (CALINE4 estimates traffic density, distance to roads), we explored the influence of geocoding accuracy by a separate analysis of the subgroup of births geocoded at the tax parcel level (the highest quality geocoding).

Because of the low percentages of missing data and long computation times, we conducted complete case analyses only. If data were missing for one case, the entire risk set it belonged to (i.e., the case and its two matched controls) was excluded from the analyses, whereas if data were missing for one control but not for the other subjects of the risk set (i.e., the matched case and other control of the risk set), these other subjects contributed to the likelihood calculation. The numbers of cases and controls included in analyses of the associations between PTB and air pollution metrics (and to some extent, the ratio of these numbers that depended on the proportion of missing data, as explained above) varied by the type of air pollution metrics because these metrics covered slightly different populations. Traffic density and distances to the nearest roadways were available for the entire state, whereas some very limited portions of the state could not be covered by the surfaces predicted by the EBK (> 98% of births were covered). CALINE4 exposures were calculated only for mothers who resided within 3 km from roadways with traffic count data (95% of all births). UCD/CIT exposures were available for the most populous area of the state where 92% of the population lived, whereas UCD_P exposures were available for the same domain, but for years 2000–2006 only.

The study has been approved by the Institutional Review Board of the University of California, Irvine. Informed consent from study participants was not required because the nature of the study was analysis of existing data, which posed minimal risk to the subjects. In addition, it was not practically feasible to contact all the subjects.

## Results

Among the eligible births, 11.43% of infants were born preterm. The distribution of cases and their matched controls by maternal characteristics, diseases, and neighborhood income level is shown in Table 1. Descriptive statistics for air pollution metrics and their correlations are presented in Table S1.

**Table 1:** Description of the case/control set population characteristics.

Population characteristic	Cases [*n* (%)]	Controls [*n* (%)]
Maternal race/ethnicity
African American	33,343 (7.54)	44,155 (4.99)
Asian	48,240 (10.91)	103,275 (11.67)
Hispanic	226,903 (51.30)	450,975 (50.98)
Non-Hispanic white	116,774 (26.40)	255,787 (28.91)
Multiple/other	10,892 (2.46)	19,928 (2.25)
Missing	6,162 (1.39)	10,508 (1.19)
Maternal education
≤ 8th grade	47,393 (10.71)	90,203 (10.20)
9th grade to high school	202,323 (45.74)	385,263 (43.55)
College (< 4 years)	87,234 (19.72)	176,389 (19.94)
College (≥ 4 years)	92,910 (21.01)	210,484 (23.79)
Missing	12,454 (2.82)	22,289 (2.52)
Median annual income by census block group
≤ $30,933	118,984 (26.90)	214,115 (24.20)
$30,938–$42,483	110,264 (24.93)	219,887 (24.86)
$42,500–$60,179	107,245 (24.25)	222,392 (25.14)
≥ $60,185	104,519 (23.63)	225,443 (25.48)
Missing	1,302 (0.29)	2,791 (0.32)
Maternal age (years)
< 15	833 (0.19)	1,032 (0.12)
15–19	44,243 (10.00)	79,909 (9.03)
20–24	94,410 (21.34)	201,263 (22.75)
25–29	105,623 (23.88)	234,977 (26.56)
30–34	105,881 (23.94)	218,580 (24.71)
35–39	69,112 (15.63)	119,637 (13.52)
40–44	19,710 (4.46)	27,485 (3.11)
45–49	2,186 (0.49)	1,599 (0.18)
≥ 50	316 (0.07)	146 (0.02)
Chronic hypertension
No	438,640 (99.17)	881,861 (99.69)
Yes	3,634 (0.82)	2,734 (0.31)
Missing	40 (0.01)	33 (0.00)
Diabetes
No	424,110 (95.88)	859,704 (97.18)
Yes	18,164 (4.11)	24,891 (2.81)
Missing	40 (0.01)	33 (0.00)
Preeclampsia
No	416,476 (94.16)	870,528 (98.41)
Yes	25,827 (5.84)	14,088 (1.59)
Missing	11 (0.00)	12 (0.00)
Primary care
First trimester	370,973 (83.87)	758,341 (85.72)
After first trimester	62,786 (14.19)	117,887 (13.33)
None	4,405 (1.00)	2,842 (0.32)
Missing	4,150 (0.94)	5,558 (0.63)
Parity
Primiparous	156,629 (35.41)	349,295 (39.48)
Multiparous	285,410 (64.53)	535,036 (60.48)
Missing	275 (0.06)	297 (0.03)
Smoking during pregnancy (2007–2008 data only)
No	110,215 (96.78)	233,461 (97.64)
Yes	3,670 (3.22)	5,651 (2.36)
Prepregnancy BMI (2007–2008 data only)
≤ 19.9	12,360 (10.85)	26,029 (10.89)
20–24.9	40,812 (35.84)	91,317 (38.19)
25–29.9	25,621 (22.50)	55,684 (23.29)
30–34.9	12,758 (11.20)	25,667 (10.73)
≥ 35	8,477 (7.44)	15,881 (6.64)
Missing	13,857 (12.17)	24,534 (10.26)

ORs for PTB in association with IQR increases in average exposure during pregnancy were positive and statistically significant: 1.133 [95% confidence interval (CI): 1.118, 1.148] for a 6.45-μg/m^3^ increase in total PM_2.5_, 1.096 (95% CI: 1.085, 1.108) for a 11.53-ppb increase in O_3_, and 1.079 (95% CI: 1.065, 1.093) for a 9.99-ppb increase in NO_2_, after adjustment for confounders (Table 2). In two-pollutant models, the positive association between PTB and PM_2.5_ was robust to adjustment for either NO_2_ or O_3_. However, the association with NO_2_ was no longer positive when adjusted for total PM_2.5_. When NO_2_ and O_3_ were both introduced into the same model, associations with PTB remained positive and significant for these two pollutants (Table 2).

**Table 2:** Associations between preterm births and measured air pollutant concentrations interpolated by empirical Bayesian kriging in California.

Air pollution indicator	Cases (*n*)	Controls (*n*)	IQR	Adjusted OR (95% CI)^*a*^	*p*-Value
Single pollutant models (years 2000–2008)
Total PM_2.5_	422,431	808,038	6.45	1.133 (1.118, 1.148)	< 0.01
O_3_	424,203	815,150	11.53	1.096 (1.085, 1.108)	< 0.01
NO_2_	421,936	806,224	9.99	1.079 (1.065, 1.093)	< 0.01
Two-pollutant model including both total PM_2.5_ and O_3_ (years 2000–2008)
Total PM_2.5_	421,068	802,401	6.45	1.120 (1.106, 1.134)	< 0.01
O_3_			11.53	1.100 (1.088, 1.112)	< 0.01
Two-pollutant model including both total PM_2.5_ and NO_2_ (years 2000–2008)
Total PM_2.5_	418,654	792,894	6.45	1.139 (1.123, 1.155)	< 0.01
NO_2_			9.99	0.986 (0.971, 1.001)	0.07
Two-pollutant model including both O_3_ and NO_2_ (years 2000–2008)
O_3_	421,597	804,812	11.53	1.096 (1.083, 1.108)	< 0.01
NO_2_			9.99	1.083 (1.069, 1.098)	< 0.01
Units are micrograms per cubic meter for total PM_2.5_, and parts per billion for gaseous pollutants. ^***a***^Odds ratios were estimated using conditional logistic regression models, adjusted for race/ethnicity and educational level using categorical variables and for maternal age and median household income at census block–group level using polynomial functions. Odds ratios are expressed per interquartile range in exposure.					

Associations between PTB and primary PM_2.5_ or PM_0.1_ modeled at a 4 km × 4 km resolution were also positive and statistically significant (Table 3). For sources of primary PM_0.1_ modeled at a 4 km × 4 km resolution using the UCD_P model (for years 2000–2006 only), the strongest associations per IQR in exposure were observed for on-road gasoline, followed by on-road diesel and commercial meat cooking. An inverse association was observed for wood burning. Patterns by source were similar for primary PM_2.5_, but overall, associations per IQR in exposure appear slightly weaker than those for PM_0.1_. However, when all sources of primary PM (including onroad gasoline, diesel, commercial meat cooking, and wood burning but also other, less well characterized sources) modeled using the UCD/CIT model for years 2000–2008 were grouped together, associations per IQR in exposure were higher for primary PM_2.5_ than for primary PM_0.1_ (Table 3).

**Table 3:** Associations between preterm births and particulate matter concentrations modeled at the 4 km × 4 km resolution by species and sources using chemical transport models in California.

Air pollution indicator	Cases (*n*)	Controls (*n*)	IQR	Adjusted OR (95% CI)^*a*^	*p*-Value
UCD/CIT modeled concentrations at the 4 km × 4 km resolution, by fraction and species (years 2000–2008)
Primary PM_0.1_	395,654	710,316	1.389	1.021 (1.015, 1.028)	< 0.01
OC in PM_0.1_	395,654	710,316	0.985	1.018 (1.012, 1.024)	< 0.01
EC in PM_0.1_	395,654	710,316	0.131	1.044 (1.036, 1.052)	< 0.01
SOA in PM_0.1_	395,654	710,316	0.061	1.130 (1.117, 1.143)	< 0.01
Primary PM_2.5_	395,654	710,316	8.229	1.036 (1.029, 1.043)	< 0.01
OC in PM_2.5_	395,655	710,316	3.699	1.020 (1.013, 1.027)	< 0.01
EC in PM_2.5_	395,654	710,316	1.258	1.040 (1.033, 1.048)	< 0.01
SOA in PM_2.5_	395,654	710,316	0.239	1.115 (1.102, 1.128)	< 0.01
Ammonium in PM_2.5_	395,654	710,316	1.188	1.138 (1.126, 1.150)	< 0.01
Nitrates in PM_2.5_	395,654	710,316	2.914	1.138 (1.128, 1.149)	< 0.01
Sulfates in PM_2.5_	395,654	710,316	0.535	1.004 (1.000, 1.008)	0.05
UCD_P modeled concentrations at the 4 km × 4 km resolution, by species, in PM_2.5_ (years 2000–2006)
Potassium	294,860	522,199	0.053	1.013 (1.003, 1.023)	< 0.01
Chromium	294,860	522,199	0.002	0.999 (0.996, 1.001)	0.31
Iron	294,860	522,199	0.190	0.980 (0.967, 0.994)	< 0.01
Titanium	294,860	522,199	0.008	0.992 (0.984, 1.001)	0.09
Magnesium	294,860	522,199	0.004	0.998 (0.990, 1.005)	0.55
Strontium	294,860	522,199	0.001	0.979 (0.969, 0.989)	< 0.01
Arsenic	294,860	522,199	0.001	0.999 (0.997, 1.000)	0.06
Calcium	294,860	522,199	0.048	0.965 (0.955, 0.975)	< 0.01
Zinc	294,860	522,199	0.002	0.982 (0.976, 0.988)	< 0.01
UCD_P modeled concentrations at the 4 km × 4 km resolution, by fraction and sources (years 2000–2006)
Onroad gasoline PM_0.1_	294,860	522,199	0.083	1.107 (1.091, 1.123)	< 0.01
Onroad diesel PM_0.1_	294,860	522,199	0.069	1.078 (1.066, 1.091)	< 0.01
Commercial meat cooking PM_0.1_	294,860	522,199	0.122	1.069 (1.058, 1.081)	< 0.01
Wood burning PM_0.1_	294,860	522,199	0.272	0.982 (0.975, 0.989)	< 0.01
Onroad gasoline PM_2.5_	294,860	522,199	0.386	1.091 (1.077, 1.106)	< 0.01
Onroad diesel PM_2.5_	294,860	522,199	0.397	1.059 (1.049, 1.070)	< 0.01
Commercial meat cooking PM_2.5_	294,860	522,199	1.084	1.041 (1.033, 1.050)	< 0.01
Wood burning PM_2.5_	294,860	522,199	1.811	0.985 (0.977, 0.993)	< 0.01
Unit is micrograms per cubic meter. ^***a***^Odds ratios were estimated using conditional logistic regression models, adjusted for race/ethnicity and educational level using categorical variables and for maternal age and median household income at census block–group level using polynomial functions. Odds ratios are expressed per interquartile range increase in exposure.					

For PM_2.5_ composition modeled at a 4 km × 4 km resolution using the UCD/CIT model (for years 2000–2008), the strongest positive associations with PTB per IQR in exposure were observed for nitrate, ammonium and SOA, followed by EC, OC, and sulfate (Table 3). For PM_2.5_ composition modeled at a 4 km × 4 km resolution using the UCD_P model (for years 2000–2006 only), a positive association was observed between PTB and potassium, whereas inverse associations were observed with iron, strontium, calcium, and zinc exposure (Table 3). No significant association was observed for the other chemical species investigated.

Analyses by quartile of exposure showed monotonic increases in PTB with increasing exposure to total PM_2.5_, O_3_, and NO_2_ estimated using EBK; and primary PM_2.5_ and PM_2.5_ species estimated using UCD/CIT; but not PM_2.5_ species estimated using UCD_P. ORs showed monotonic increases across quartiles of primary PM_0.1_ and PM_0.1_ species estimated using UCD/CIT; and for UCD_P estimates of primary PM (either PM_2.5_ or PM_0.1_) from on-road gasoline, on-road diesel, and commercial meat cooking. Nevertheless, ORs for primary PM (either PM_2.5_ or PM_0.1_) from wood burning decreased as exposures increased (see Figure S2).

For indicators of traffic-related pollution at fine geographical resolution (CALINE4 predictions, traffic density, and distance to roads), associations with PTB were sensitive to the accuracy of geocoding. In the entire population, these indicators were generally inversely associated with PTB (Table 4). However when analyses were restricted to the births geocoded at the tax parcel level, CALINE4 predictions for UFP, CO, and NO_x_ were all positively associated with PTB (Table 4). Positive associations with traffic density and distance to roads (only within 150 m for distance to roads) were also observed only when restricting to parcel geocoded births (Table 4).

**Table 4:** Associations between preterm births and indicators of traffic-related pollution at fine geographic scale in California, by geocoding accuracy.

Air pollution indicator	All subjects (regardless of geocoding accuracy of maternal residences)					Subjects with maternal residences geocoded at the tax parcel level				
	Cases (*n*)	Controls (*n*)	IQR	Adjusted OR (95% CI)^*a*^	*p*-Value	Cases (*n*)	Controls (*n*)	IQR	Adjusted OR (95% CI)^*a*^	*p*-Value
CALINE4 modeled concentrations (years 2000–2008)
UFP	397,047	741,023	6,480	0.994 (0.988, 1.000)	0.04	212,010	216,885	6,770	1.028 (1.021, 1.036)	< 0.01
CO	397,047	741,023	58.79	1.003 (0.996, 1.009)	0.44	212,010	216,885	64.65	1.044 (1.035, 1.053)	< 0.01
NO_x_	397,047	741,023	5.97	1.005 (0.999, 1.011)	0.08	212,010	216,885	6.47	1.034 (1.026, 1.042)	< 0.01
Traffic density (within buffers of different sizes, years 2000–2008)
50-m buffer	427,642	829,442		0.968 (0.940, 0.996)	0.03	232,775	250,195		1.063 (1.013, 1.117)	0.01
150-m buffer	427,642	829,442		0.987 (0.972, 1.002)	0.09	232,775	250,195		1.048 (1.026, 1.072)	< 0.01
250-m buffer	427,642	829,442		0.964 (0.950, 0.979)	< 0.01	232,775	250,195		1.011 (0.990, 1.033)	0.29
350-m buffer	427,642	829,442		0.962 (0.947, 0.978)	< 0.01	232,775	250,195		1.012 (0.989, 1.035)	0.31
Distance to roadways (years 2000–2008)
< 50 m	427,762	829,871		0.983 (0.975, 0.991)	< 0.01	232,863	250,398		0.996 (0.983, 1.009)	0.54
< 100 m	427,762	829,871		0.991 (0.984, 0.998)	< 0.01	232,863	250,398		1.009 (0.998, 1.020)	0.11
< 150 m	427,762	829,871		0.988 (0.980, 0.996)	0.01	232,863	250,398		1.013 (1.002, 1.023)	0.02
< 200 m	427,762	829,871		0.981 (0.973, 0.990)	< 0.01	232,863	250,398		1.003 (0.992, 1.014)	0.63
Unit is parts per billion for gaseous pollutants. ^***a***^Odds ratios were estimated using conditional logistic regression models, adjusted for race/ethnicity and educational level using categorical variables and for maternal age and median household income at census block–group level using polynomial functions. For estimated pollutant concentrations, odds ratios are expressed per interquartile range. For traffic density, they are expressed per 10,000 vehicles per day per meter. For distance to roadways, they compare births within the stated distance to those outside that distance.										

Sensitivity analyses showed that further adjustment for PTB risk factors other than maternal age, race/ethnicity, education, and neighborhood median income changed risk estimates by ≤ 10%, except for BMI. The results of sensitivity analyses (years 2007–2008) with adjustment for BMI or smoking, in addition to the covariates included in the primary models are shown in Table S2.

Overall, similar results were observed for MPTB and VPTB as those for PTB, except positive associations were observed between MPTB and iron, titanium, magnesium and strontium and between VPTB and titanium and magnesium (see Table S3). However, no significant association was observed between VPTB and UCD_P predictions of PM by sources.

## Discussion

A major asset of this large study is the wealth of air pollution metrics. California has the densest ambient PM measurement network of any state in the United States, and detailed emissions inventories (Hu et al. 2015). Rich environmental data sets (e.g., PM species measurements and receptor-oriented source apportionment studies at multiple sites) were available to support exposure model application and evaluation.

The respective strengths and limitations of the various air pollution metrics used in this study, notably for their use in epidemiological studies of pregnancy outcomes, have been discussed extensively in other papers (Benson 1989; Hu et al. 2014a, 2014b, 2015; Laurent et al. 2013, 2014; Wu et al. 2009b) and in a report (Wu et al. 2016). Briefly, the interpolation of ambient measurements for PM_2.5_, NO_2_, and O_3_ using EBK avoids biases from assigning data from one single monitor to populations living farther away (Laurent et al. 2014). It captures general temporal and spatial trends in ambient concentrations of three pollutants (total PM_2.5_, NO_2_, and O_3_), but not the small-scale spatial variations (e.g., within a few hundred meters) because only 75–182 monitoring sites were located over the entire state of California depending on the pollutant and time period, and because EBK does not incorporate spatial covariates for prediction (in contrast with land use regression). However, leave-one-out cross-validation results for EBK were satisfactory for monthly concentrations, with correlation coefficients of 0.74, 0.72, and 0.65 for O_3_, NO_2_, and total PM_2.5_ respectively (Wu et al. 2016).

The chemical transport models capture spatial variability in ambient concentrations better, but are less capable at capturing temporal variability. However, they cover pollutants for which measurement data are very scarce, such as ultrafine PM (Hu et al. 2014b), chemical species in PM (Hu et al. 2014a, 2015), and source-specific primary PM (Hu et al. 2014b). Validation studies have been conducted for UCD_P (Hu et al. 2014a, 2014b) and UCD/CIT (Hu et al. 2015) to identify those particle size fractions, chemical components, and sources that are suitable for inclusion in epidemiologic studies. Only four major sources of primary PM that passed the direct validation checks (agreement between modeled concentrations and the results of source apportionment studies across several locations and in different episodes in California, as explained above and in “Description of the source constraint checks performed to directly evaluate the accuracy of simulated source contributions using the UCD_P model” in the Supplemental Material) were included in the present study. They represent some of the most ubiquitous sources in the environment of urban and/or rural populations (Hu et al. 2014b). Similarly, we included in the analyses only primary PM components for which correlations between monthly averaged predictions and measured values were > 0.8. Total PM_0.1_ mass was also included in the analysis because prediction agreed well with measurements (*R* = 0.81) (Hu et al. 2014a). For secondary species, we included pollutants with model performance in reasonably good agreement with measurements (for concentrations averaged on several months: organic carbon, nitrate, and ammonium), according to standard criteria for acceptable model performance as discussed by Boylan and Russell (2006): mean fractional error ≤ 50% and mean fractional bias within ± 30% (Hu et al. 2015). Sulfates were also included because they are a non-negligible contributor to total PM mass (Bell et al. 2007) even though the predicted sulfate concentrations are not satisfactory (Boylan and Russell 2006) due to missing emission sources (Hu et al. 2015). Predictions for SOA concentrations could not be validated because it is difficult to differentiate the SOA fraction from total organic aerosol in the measurements. Caution must therefore be taken when interpreting results for sulfate and SOA in our study.

Because both EBK and chemical transport models had limited geographical resolution and traffic emissions may be highly heterogeneous at finer geographic scales, CALINE4 predictions were used to capture such small-scale variations in primary traffic emissions (Benson 1989; Laurent et al. 2013; Wu et al. 2009a). CALINE4 estimates have limited temporal variability because CALINE4 is a simple Gaussian dispersion model that does not consider complex atmospheric mechanisms of transport, deposition, chemical reaction, and gas-particle transformation. In addition, model inputs have limited temporal resolution (e.g., annual average traffic counts, estimated mixing height by season and time of day). However, the model worked reasonably well with an overall correlation of 0.75 between modeled and measured daily average particle number concentrations at three monitoring sites in Los Angeles County and one site in Riverside County, California (Wu et al. 2016).

Traffic density and distance to roads are cruder proxies of traffic-related pollution than CALINE4 predictions, but were used to check for consistency of our results with numerous studies which used similar indicators.

As a general limitation, the personal exposure of mothers during pregnancy could not be estimated in this study because we did not have time–activity information for the population (Wu et al. 2011). Our air pollution metrics relied solely on maternal home address at the time of delivery because previous residences during pregnancy were unavailable in birth certificates.

The statistical models used for the main analyses were adjusted for a set of potential confounders selected using a causal diagram: maternal age, race/ethnicity, education, and neighborhood median income. All of them are strong and well-documented risk factors for PTB and air pollution exposures, and they are reported with relatively high accuracy on birth certificates (Northam and Knapp 2006). Additional adjustment for further risk factors had very limited impact on the results; therefore, results of such analyses are provided only for smoking or BMI (see Table S2). It is possible that the negligible impact of adjusting for smoking or for some chronic diseases is partly attributable to underreporting of these factors in birth certificates, leading to a very low proportion of women with such factors effectively documented in our population (0.4% for chronic hypertension, 2.9% for total diabetes, and 2.5% for smoking in 2007–2008 data). However, our sensitivity analyses suggest that if further adjusting for smoking has any effect, it is toward increasing the risk estimates of the associations between air pollution and PTB (except for O_3_). On the contrary, further adjusting for BMI slightly reduced the associations between most air pollutants and PTB, but did not change the conclusions of the analyses (see Table S2).

We found a positive association between PTB and total measured PM_2.5_. This is consistent with some (Dadvand et al. 2014; Kloog et al. 2012; Pereira et al. 2014; Stieb et al. 2012) but not all (Trasande et al. 2013; Wilhelm et al. 2011) previous studies. Again, because the composition of PM_2.5_ substantially varies in time and space, contrasted findings from one setting to another are expected. Our PM_2.5_ finding is consistent with a previous California study conducted during an earlier period (1999–2000) (Huynh et al. 2006).

Only previous studies of smaller sizes (i.e., including ≤ 50,000 PTB cases) examined the associations between PTB and PM by source (Brauer et al. 2008; Généreux et al. 2008; Huppé et al. 2013; Malmqvist et al. 2011; Miranda et al. 2013; Mohorovic 2004; Parker et al. 2008; Wilhelm et al. 2011; Wu et al. 2009b; Wylie et al. 2014; Yang et al. 2003, 2004; Yorifuji et al. 2011) or composition (Darrow et al. 2009; Wilhelm et al. 2011). We found primary PM from several sources to be positively associated with PTB. Consistent findings have been reported previously from other studies for diesel sources and meat cooking (Wilhelm et al. 2011).

To the best of our knowledge, this is the first study of ultrafine particles and PTB. Positive associations were observed with both the mass (UCD_P) and number (CALINE4, in births geocoded to tax parcels) of primary ultrafine particles (from local traffic emissions, for the latter). In our study associations between PTB and an IQR increase in PM from each source were slightly stronger for PM_0.1_ than for PM_2.5_, as expected because of the higher total surface area available for adsorption of toxic chemicals and higher potential for translocation of PM_0.1_, than of PM_2.5_ (Knol et al. 2009). However, an opposite pattern (stronger association with an IQR increase in PM_2.5_ compared with PM_0.1_) was observed for the total mass of primary PM, which was unexpected for the reasons mentioned above. The observed inverse associations between PTB and PM_0.1_ or PM_2.5_ from wood burning were also unexpected.

Our finding of an association with EC and OC in PM is consistent with a previous study based on speciation monitor measurements in Los Angeles (Wilhelm et al. 2011). The positive associations observed with NO_2_ (EBK) and NO_x_ (CALINE4, in births geocoded to tax parcels) are also echoed by other studies (Dadvand et al. 2014; Wilhelm et al. 2011). The associations we observed for potassium (positive), strontium, zinc, iron, and calcium (all inverse) are unprecedented, but no clear dose–response gradients were observed for these species, except for calcium (see Figure S2).

Regarding secondary pollutants, we found an increased risk of PTB with exposure to O_3_ [consistent with some (Lin et al. 2015; Olsson et al. 2013) but not all (Stieb et al. 2012) previous studies] and other secondary pollutants such as nitrate, ammonium, SOA, and sulfate. A recent study in Los Angeles County reported a positive association between PTB and measured ammonium nitrate in PM_2.5_ (Wilhelm et al. 2011). This is clearly consistent with our results. However, another study in Atlanta using a time-series methodology reported no association between PTB and nitrate or ammonium (Darrow et al. 2009). Darrow et al. (2009) also reported a positive association with sulfate, a pollutant that has higher concentrations in the eastern than in western United States (Bell et al. 2007) and for which the modeling performance was suboptimal in our study setting (Hu et al. 2015). We estimated a novel positive association between PTB and SOA, but this finding should be interpreted with caution given that SOA predictions have not been validated against measured values.

Interestingly, our findings for the associations between PTB and local traffic-related pollution characterized at a fine geographical resolution using predictions from the CALINE4 model, traffic density, or distance to roads are highly sensitive to the accuracy of geocoding. If an increase in PTB risk really occurs only within a short distance from roads (e.g., < 200 m), as our results suggest, then imprecise geocoding could easily introduce substantial exposure measurement error, obscuring any epidemiological associations with local traffic emissions. This might help explain some inconsistent findings from the literature, although most studies have reported positive associations between PTB and traffic exposure (Généreux et al. 2008; Miranda et al. 2013; Wu et al. 2009b; Yorifuji et al. 2011). Future studies may benefit from restriction to participants with the highest quality geocode, at least as part of sensitivity analyses.

## Conclusions

In this large study, both primary and secondary pollutants are associated with increased PTB risk. Consistent results obtained using complementary exposure matrices supports previous evidence that primary traffic-related pollutants might increase PTB risk. Among the sources of primary PM_0.1_ and PM_2.5_ we evaluated, traffic (as represented by on-road gasoline and diesel) is the most strongly associated with PTB per IQR in exposure. Positive associations between PTB and PM_2.5_ components were the strongest for IQR increases in nitrate, ammonium and secondary organic aerosols, followed by elemental carbon and organic carbon.

## Supplemental Material

(570 KB) PDF
