# Supplementary material for: A Statewide Nested Case–Control Study of Preterm Birth and Air Pollution by Source and Composition: California, 2001–2008
Source: Environ Health Perspect. 2016 Feb 19;124(9):1479–86. doi: 10.1289/ehp.1510133 (PMC5010414; doi:10.1289/ehp.1510133)
Supplement: (570 KB) PDF [file ehp.1510133.s001.acco.pdf]

Environ Health Perspect
DOI: 10.1289/ehp.1510133

Note to readers with disabilities: *EHP* strives to ensure that all journal content is accessible to all readers. However, some figures and Supplemental Material published in *EHP* articles may not conform to 508 standards due to the complexity of the information being presented. If you need assistance accessing journal content, please contact ehp508@niehs.nih.gov. Our staff will work with you to assess and meet your accessibility needs within 3 working days.

Supplemental Material

A Statewide Nested Case-Control Study of Preterm Birth and Air Pollution by Source and Composition: California, 2001–2008

Olivier Laurent, Jianlin Hu, Lianfa Li, Michael J. Kleeman, Scott M. Bartell, Myles Cockburn, Loraine Escobedo, and Jun Wu

Table of Contents

Description of the source constraint checks performed to directly evaluate the accuracy of simulated source contributions using the UCD_P model

Figure S1. Directed acyclic graph of assumed relationships between preterm birth, air pollution and other risk factors, based on literature data.

Table S1. Correlation matrix for pollutants

Figure S2. Odds ratios of preterm birth by quartile of air pollution exposure

Table S2. Sensitivity analysis of preterm birth and air pollution, by adjustment for smoking or body mass index, in addition to the covariates included in the primary models (years 2007-2008)

Table S3. Sensitivity analysis of moderately preterm birth (MPTB, gestational age <35 weeks) or very preterm birth (VPTB, gestational age <30 weeks) and air pollution

References

Description of the source constraint checks performed to directly evaluate the accuracy of simulated source contributions using the UCD_P model.

The accuracy of estimates for PM contributions from major sources was evaluated by comparison to receptor-oriented source apportionment calculations based on measurements of PM molecular marker concentrations during specialized field campaigns. Although such campaigns do not occur frequently, good agreement with the available data does build confidence in predictions of PM source contributions from major categories including mobile sources, food cooking, and wood burning. The accuracy of contributions from other minor sources was evaluated by comparison to routine measurements of less-specific component concentrations (elemental carbon/organic carbon/metals). For each component that was predicted accurately at a measurement site by the model (correlation ≥ 0.8 and mean fractional bias within ± 0.3), the top 95% of sources contributing to that component concentration within 100 km of the measurement site were identified. Sources identified through this procedure at 3 or more measurement sites were judged to be accurately predicted since their concentrations were consistent with available measurements. (Hu et al. 2014).

Figure S1. Directed acyclic graph of assumed relationships between preterm birth, air pollution and other risk factors, based on literature data.

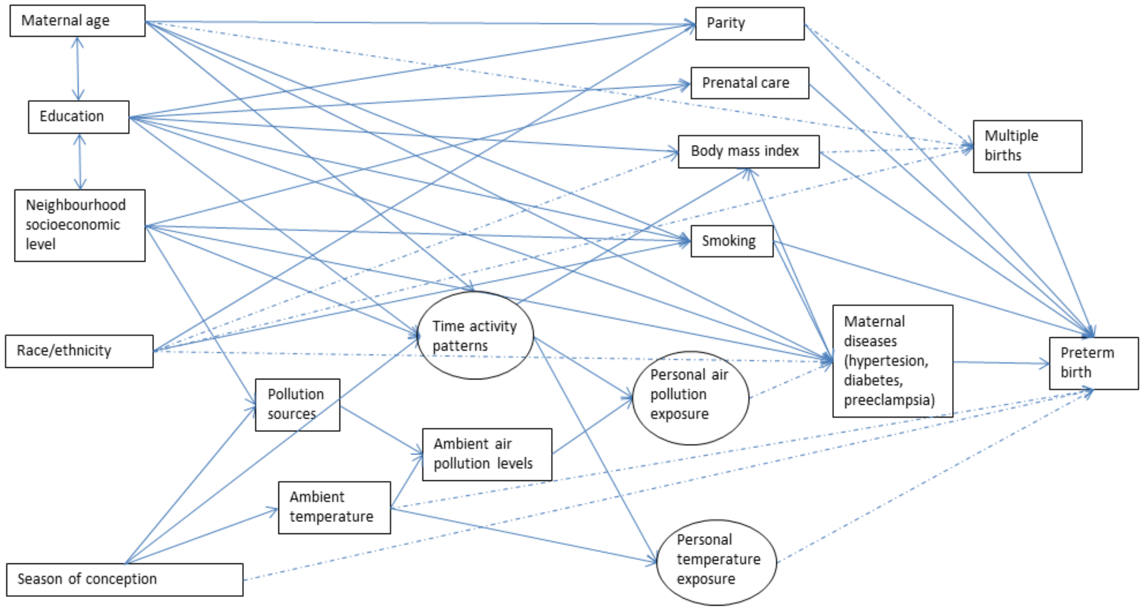

Plain arrows represent established relationships. Dotted arrows represent relationships for which a greater degree of uncertainty exists (observations based on a few studies and that would call for confirmation by more studies, and/or associations for which mechanisms are not well understood). Square represent observed variables and circles unobserved variables.

The causal diagram theory (Greenland et al. 1999) warns against adjusting for potential colliders, which are factors determined by two or more other factors already included in the model (e.g., parity, which is determined in part by maternal age and socioeconomic factors) in order to avoid over-adjustment bias. Based on the causal diagram above, the statistical models we used for the primary analyses were adjusted for a minimal sufficient set of potential confounders, namely maternal age, race/ethnicity, education, and neighborhood socioeconomic level.

Table S1. Correlation matrix for pollutants[a].

Variable[b]	Mean	Std Dev	EBK $PM_{2.5}$	EBK O_3	EBK NO_2	Primary $PM_{0.1}$	OC in $PM_{0.1}$	EC in $PM_{0.1}$	SOA in $PM_{0.1}$	Primary $PM_{2.5}$	OC in $PM_{2.5}$	EC in $PM_{2.5}$	SOA in $PM_{2.5}$	Ammonium in $PM_{2.5}$	Nitrates in $PM_{2.5}$	Sulfates in $PM_{2.5}$	Arsenic in $PM_{2.5}$	Calcium in $PM_{2.5}$	Chromium in $PM_{2.5}$	Iron in $PM_{2.5}$	Potassium in $PM_{2.5}$	Magnesium in $PM_{2.5}$	Strontium in $PM_{2.5}$	Titanium in $PM_{2.5}$	Zinc in $PM_{2.5}$	Onroad gasoline $PM_{0.1}$	Onroad diesel $PM_{0.1}$	Meat cooking $PM_{0.1}$	Wood burning $PM_{0.1}$	Onroad gasoline $PM_{2.5}$	Onroad diesel $PM_{2.5}$	Meat cooking $PM_{2.5}$	Wood burning $PM_{2.5}$	CALINE UFP number	CALINE CO
**EBK $PM_{2.5}$**	14.39	4.43																																	
**EBK O_3**	39.71	8.66	0.09																																
**EBK NO_2**	19.15	6.75	0.81	-0.07																															
**Primary $PM_{0.1}$**	1.74	1.05	0.40	-0.24	0.43																														
**OC in $PM_{0.1}$**	1.24	0.78	0.38	-0.23	0.39	0.99																													
**EC in $PM_{0.1}$**	0.16	0.09	0.48	-0.19	0.64	0.70	0.66																												
**SOA in $PM_{0.1}$**	0.07	0.04	0.59	0.63	0.42	0.24	0.22	0.31																											
**Primary $PM_{2.5}$**	14.83	5.79	0.44	-0.28	0.44	0.82	0.80	0.81	0.27																										
**OC in $PM_{2.5}$**	5.08	2.63	0.36	-0.36	0.42	0.89	0.88	0.77	0.12	0.92																									
**EC in $PM_{2.5}$**	1.55	0.88	0.47	-0.21	0.63	0.69	0.64	0.99	0.28	0.81	0.76																								
**SOA in $PM_{2.5}$**	0.32	0.16	0.50	0.59	0.33	0.19	0.17	0.29	0.93	0.32	0.12	0.28																							
**Ammonium in $PM_{2.5}$**	1.43	0.80	0.71	0.31	0.50	0.39	0.35	0.44	0.76	0.44	0.28	0.42	0.68																						
**Nitrates in $PM_{2.5}$**	3.25	1.98	0.70	0.32	0.46	0.29	0.27	0.41	0.71	0.37	0.24	0.39	0.59	0.95																					
**Sulfates in $PM_{2.5}$**	0.91	0.73	0.33	-0.13	0.35	0.57	0.52	0.35	0.20	0.45	0.38	0.36	0.24	0.40	0.15																				
**Arsenic in $PM_{2.5}$**	0.001	0.003	0.00	-0.18	0.03	0.04	0.03	0.03	-0.10	0.12	0.07	0.04	-0.08	-0.03	-0.05	0.21																			
**Calcium in $PM_{2.5}$**	0.067	0.037	-0.13	-0.33	-0.20	0.39	0.38	0.11	-0.19	0.46	0.39	0.11	-0.15	-0.05	-0.10	0.24	0.07																		
**Chromium in $PM_{2.5}$**	0.003	0.003	0.12	-0.22	0.14	0.30	0.28	0.18	-0.03	0.33	0.30	0.18	-0.02	0.12	0.03	0.48	0.72	0.29																	
**Iron in $PM_{2.5}$**	0.238	0.132	-0.13	-0.21	-0.26	0.26	0.28	0.08	-0.12	0.43	0.33	0.07	-0.06	-0.01	0.01	-0.06	0.08	0.85	0.26																
**Potassium in $PM_{2.5}$**	0.081	0.047	0.04	-0.07	-0.18	0.26	0.28	0.04	0.04	0.38	0.33	0.02	0.04	0.19	0.25	-0.16	0.04	0.65	0.16	0.85															
**Magnesium in $PM_{2.5}$**	0.005	0.003	0.01	-0.16	-0.10	0.26	0.26	0.10	-0.01	0.37	0.26	0.09	0.02	0.11	0.10	0.07	0.12	0.66	0.36	0.80	0.67														
**Strontium in $PM_{2.5}$**	0.001	0.001	-0.07	-0.38	-0.09	0.42	0.42	0.26	-0.19	0.56	0.50	0.26	-0.14	-0.05	-0.07	0.16	0.07	0.90	0.26	0.88	0.64	0.64													
**Titanium in $PM_{2.5}$**	0.011	0.006	0.01	-0.17	-0.10	0.34	0.34	0.19	-0.02	0.46	0.37	0.19	0.02	0.11	0.11	0.10	0.04	0.73	0.21	0.81	0.69	0.63	0.77												
**Zinc in $PM_{2.5}$**	0.004	0.003	-0.03	-0.34	-0.03	0.36	0.34	0.15	-0.17	0.40	0.36	0.16	-0.14	0.00	-0.07	0.42	0.62	0.53	0.73	0.42	0.27	0.36	0.53	0.40											

Table S1 (continued). Correlation matrix for pollutants[a]

Variable[b]	Mean	Std Dev	EBK $PM_{2.5}$	EBK O_3	EBK NO_2	Primary $PM_{0.1}$	OC in $PM_{0.1}$	EC in $PM_{0.1}$	SOA in $PM_{0.1}$	Primary $PM_{2.5}$	OC in $PM_{2.5}$	EC in $PM_{2.5}$	SOA in $PM_{2.5}$	Ammonium in $PM_{2.5}$	Nitrates in $PM_{2.5}$	Sulfates in $PM_{2.5}$	Arsenic in PM2.5	Calcium in PM2.5	Chromium in PM2.5	Iron in PM2.5	Potassium in PM2.5	Magnesium in PM2.5	Strontium in PM2.5	Titanium in PM2.5	Zinc in PM2.5	Onroad gasoline $PM_{0.1}$	Onroad diesel $PM_{0.1}$	Meat cooking $PM_{0.1}$	Wood burning $PM_{0.1}$	Onroad gasoline $PM_{2.5}$	Onroad diesel $PM_{2.5}$	Meat cooking $PM_{2.5}$	Wood burning $PM_{2.5}$	CALINE UFP number	CALINE CO
**Onroad gasoline $PM_{0.1}$**	0.06	0.05	0.59	-0.11	0.77	0.58	0.54	0.83	0.35	0.61	0.61	0.83	0.27	0.43	0.41	0.39	0.04	-0.15	0.12	-0.25	-0.26	-0.14	-0.03	-0.07	0.04										
**Onroad diesel $PM_{0.1}$**	0.06	0.04	0.55	-0.15	0.71	0.66	0.62	0.87	0.33	0.69	0.70	0.88	0.25	0.43	0.41	0.38	0.04	-0.04	0.16	-0.12	-0.14	-0.05	0.09	0.03	0.10	0.95									
**Meat cooking $PM_{0.1}$**	0.10	0.09	0.61	-0.04	0.73	0.56	0.52	0.82	0.37	0.59	0.63	0.81	0.31	0.46	0.44	0.33	-0.02	-0.13	0.13	-0.17	-0.15	-0.09	0.04	0.05	0.00	0.84	0.84								
**Wood burning $PM_{0.1}$**	0.27	0.32	-0.14	-0.36	-0.17	0.44	0.47	0.10	-0.26	0.43	0.59	0.08	-0.28	-0.17	-0.12	-0.12	0.02	0.49	0.13	0.50	0.60	0.31	0.50	0.35	0.34	-0.09	0.03	-0.08							
**Onroad gasoline $PM_{2.5}$**	0.35	0.24	0.56	-0.17	0.74	0.61	0.57	0.85	0.31	0.66	0.66	0.86	0.24	0.39	0.38	0.39	0.05	-0.04	0.15	-0.14	-0.19	-0.07	0.09	0.02	0.10	0.99	0.96	0.83	-0.03						
**Onroad diesel $PM_{2.5}$**	0.45	0.27	0.48	-0.22	0.62	0.70	0.66	0.88	0.27	0.77	0.77	0.88	0.21	0.39	0.38	0.35	0.06	0.16	0.21	0.09	0.03	0.11	0.29	0.19	0.20	0.87	0.96	0.79	0.14	0.91					
**Meat cooking $PM_{2.5}$**	1.07	0.81	0.43	-0.19	0.54	0.56	0.52	0.78	0.19	0.67	0.72	0.76	0.16	0.30	0.29	0.25	0.02	0.18	0.24	0.15	0.08	0.12	0.37	0.27	0.17	0.66	0.72	0.88	0.15	0.70	0.77				
**Wood burning $PM_{2.5}$**	1.76	1.91	-0.13	-0.32	-0.23	0.39	0.43	0.04	-0.23	0.40	0.53	0.02	-0.24	-0.11	-0.05	-0.15	0.02	0.55	0.11	0.61	0.74	0.40	0.54	0.45	0.34	-0.20	-0.07	-0.16	0.97	-0.13	0.07	0.09			
**CALINE UFP number**	6111	5894	0.23	-0.18	0.36	0.36	0.34	0.47	0.08	0.41	0.41	0.47	0.06	0.10	0.09	0.23	0.03	0.06	0.08	-0.02	-0.11	-0.02	0.14	0.03	0.13	0.53	0.53	0.42	0.05	0.55	0.52	0.39	-0.01		
**CALINE CO**	58.75	50.87	0.35	-0.22	0.46	0.38	0.37	0.45	0.11	0.43	0.43	0.45	0.11	0.10	0.08	0.25	0.04	0.09	0.11	0.01	-0.09	0.01	0.19	0.08	0.14	0.53	0.52	0.49	0.06	0.55	0.52	0.49	-0.01	0.89	
**CALINE NO_x**	6.10	5.48	0.35	-0.19	0.43	0.38	0.36	0.43	0.12	0.41	0.40	0.42	0.10	0.14	0.12	0.26	0.04	0.10	0.13	0.04	-0.04	0.05	0.18	0.09	0.15	0.48	0.49	0.43	0.06	0.50	0.49	0.42	0.01	0.91	0.94

a) Based on entire pregnancy exposure.

b) Units are micrograms per cubic meter for all particulate mass and elements, part per billion for gaseous pollutants; EBK : empirical Bayesian krigining. $PM_{2.5}$; particulate matter less than 2.5 µm in aerodynamic diameter; O_3: ozone; NO_2: nitrogen dioxide; $PM_{0.1}$; particulate matter less than 0.1 µm in aerodynamic diameter; EC: elemental carbon ; OC: organic carbon; SOA secondary organic aerosols; UFP: ultrafine particles; CO: carbon monoxide; NO_x: nitrogen oxides.

Figure S2. Odds ratios of preterm birth by quartile of air pollution exposure.

For each pollutant, the quartiles of exposure (averaged from the day of conception to the delivery date of the case in each case control set, see material and methods section) are ranked from left (first quartile) to right (fourth quartile). Dots with bars represent odds ratios for preterm birth and associated 95% confidence intervals in the second, third and fourth quartiles of exposure as compared to the first quartile of exposure (reference group, dot without bar).

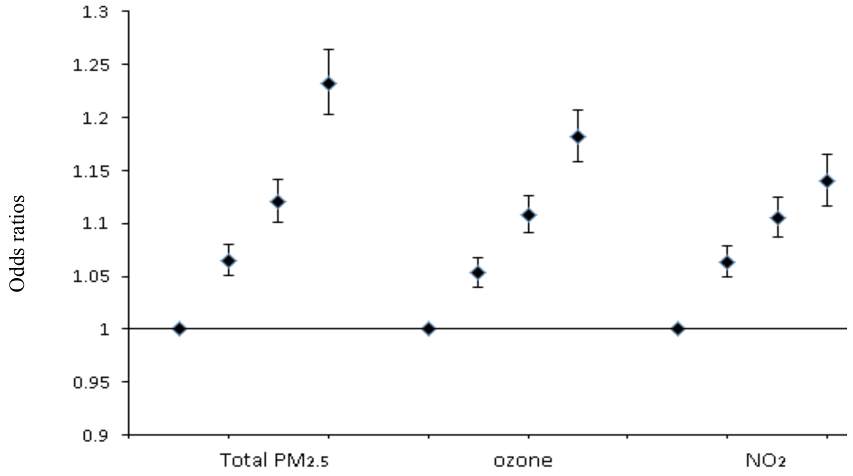

Measured pollutant concentrations interpolated by empirical Bayesian kriging (years 2000-2008).

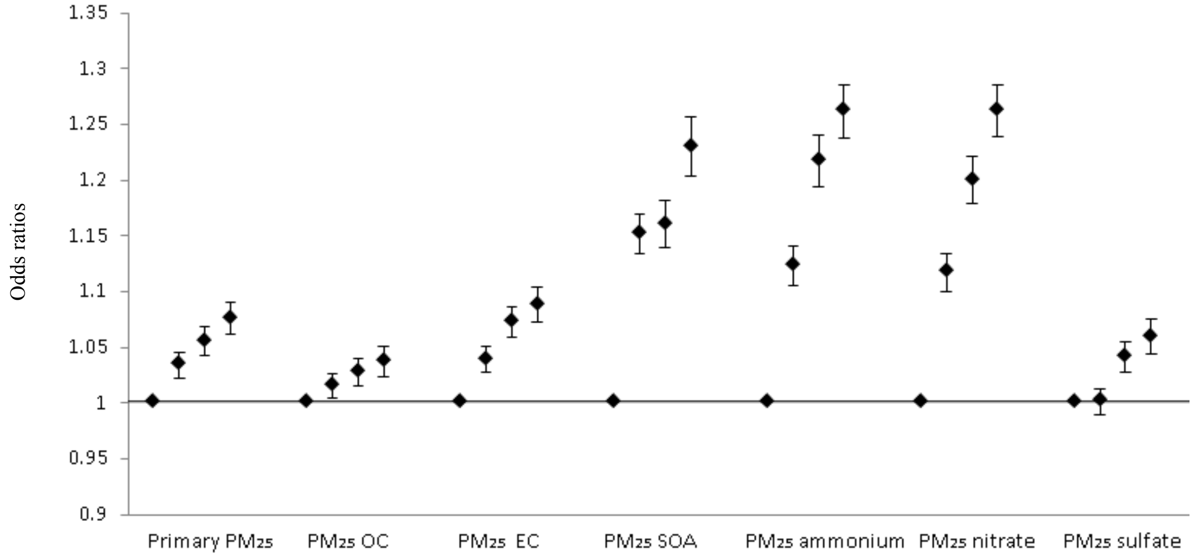

Concentrations of primary $PM_{2.5}$ and of species in $PM_{2.5,}$ modeled at the 4 km*4 km resolution using the UCD_CIT chemical transport model (years 2000-2008).

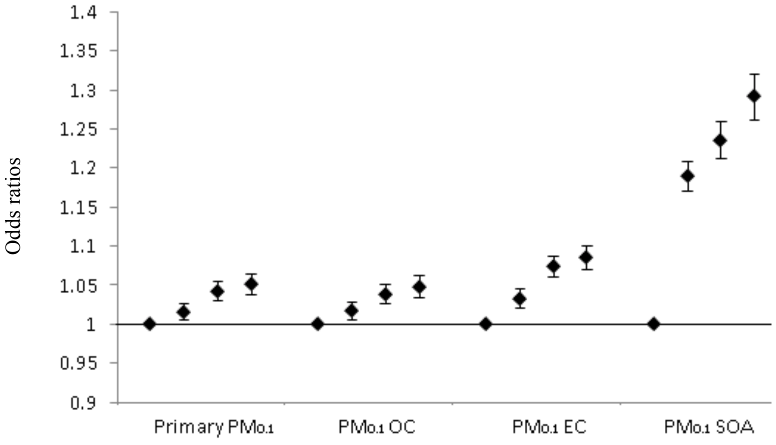

Concentrations of primary $PM_{0.1}$ and of species in $PM_{0.1}$, modeled at the 4 km*4 km resolution using the UCD_CIT chemical transport model (years 2000-2008).

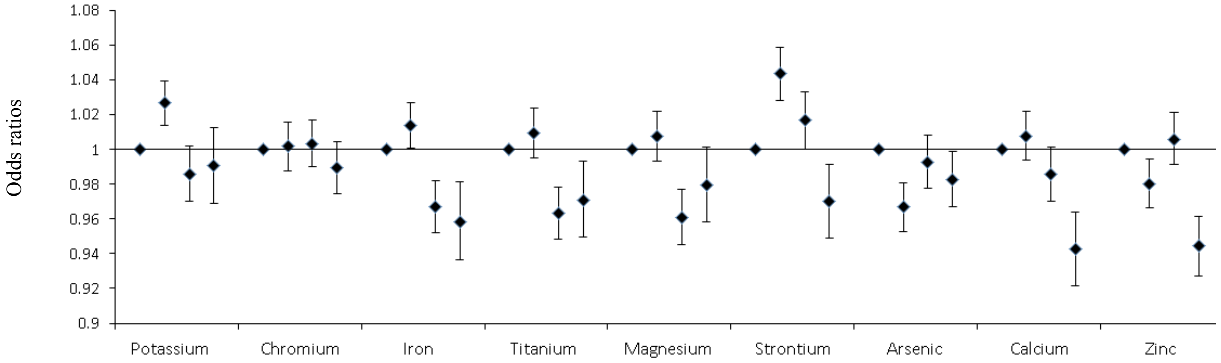

Concentrations of species in $PM_{2.5}$, modeled at the 4 km*4 km resolution using the UCD_P chemical transport model (years 2000-2006).

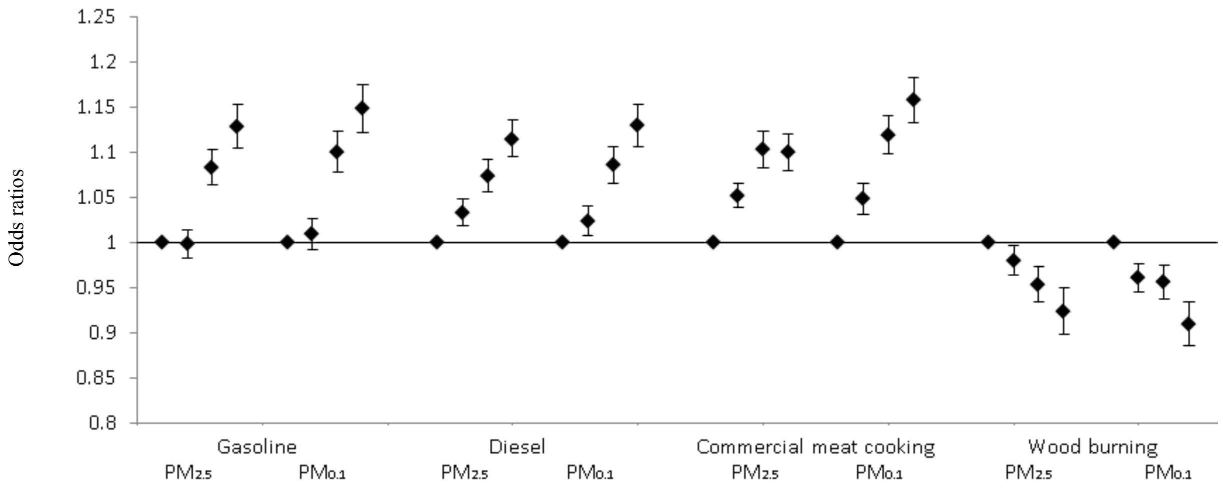

Concentrations of primary $PM_{2.5}$ and $PM_{0.1}$ mass by source, modeled at the 4 km*4 km resolution using the UCD_P chemical transport model (years 2000-2006).

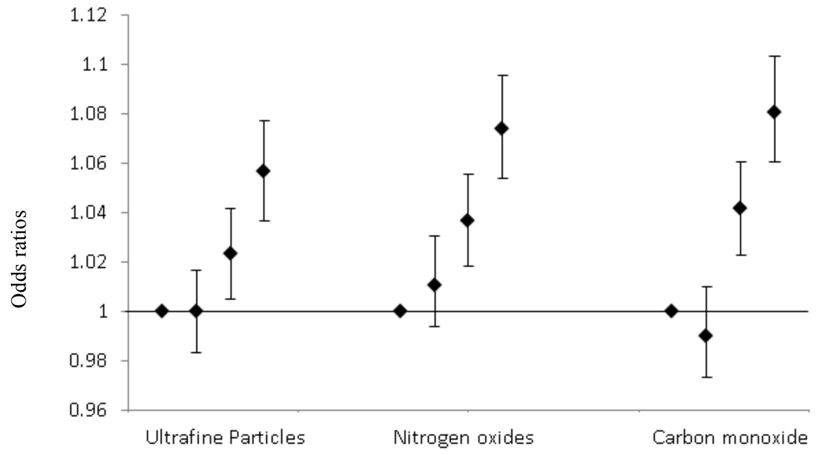

Concentrations of primary pollutants from local traffic modelled with CALINE4, for infants with maternal addresses geocoded to the parcel level (years 2000-2008).

Table S2. Sensitivity analysis of preterm birth and air pollution, by adjustment for smoking or body mass index, in addition to the covariates included in the primary models (years 2007-2008).

Air pollution indicator[a]	Number of cases	Number of controls	IQR[b]	Primary models[c]	Primary models[c], plus smoking[d]	Primary models[c], plus body mass index[d]
				Adjusted odd ratio per IQR[b] (95% confidence interval)	Adjusted odd ratio per IQR[b] (95% confidence interval)	Adjusted odd ratio per IQR[b] (95% confidence interval)
**Measured pollutant concentrations interpolated by empirical Bayesian kriging**						
$PM_{2.5}$	107,830	205,045	4.72	1.128 (1.114, 1.142)	1.130 (1.116, 1.144)	1.114 (1.099, 1.129)
O_3	108,261	206,793	10.45	1.037 (1.025, 1.049)	1.035 (1.023, 1.047)	1.053 (1.040, 1.067)
NO_2	108,092	206,204	8.91	1.101 (1.086, 1.117)	1.105 (1.090, 1.121)	1.088 (1.071, 1.104)
**UCD_CIT modeled concentrations at the 4km*4km resolution. by fraction and species**						
Primary $PM_{0.1}$	100,794	179,198	1.47	1.073 (1.063, 1.084)	1.074 (1.064, 1.085)	1.069 (1.057, 1.081)
OC in $PM_{0.1}$	100,794	179,198	1.04	1.069 (1.059, 1.079)	1.070 (1.060, 1.080)	1.067 (1.056, 1.079)
EC in $PM_{0.1}$	100,794	179,198	0.15	1.060 (1.047, 1.073)	1.063 (1.050, 1.076)	1.045 (1.032, 1.059)
SOA in $PM_{0.1}$	100,794	179,198	0.05	1.029 (1.015, 1.044)	1.031 (1.016, 1.045)	1.031 (1.015, 1.046)
Primary $PM_{2.5}$	100,794	179,198	10.01	1.080 (1.066, 1.095)	1.082 (1.068, 1.097)	1.069 (1.054, 1.084)
OC in $PM_{2.5}$	100,794	179,198	4.10	1.076 (1.064, 1.088)	1.077 (1.065, 1.089)	1.069 (1.056, 1.082)
EC in $PM_{2.5}$	100,794	179,198	1.48	1.054 (1.041, 1.067)	1.057 (1.044, 1.070)	1.037 (1.024, 1.050)
SOA in $PM_{2.5}$	100,794	179,198	0.26	0.959 (0.941, 0.977)	0.961 (0.943, 0.979)	0.960 (0.941, 0.979)
Ammonium in $PM_{2.5}$	100,794	179,198	1.22	1.174 (1.159, 1.190)	1.176 (1.160, 1.191)	1.168 (1.153, 1.185)

Air pollution indicator[a]	Number of cases	Number of controls	IQR[b]	Primary models[c]	Primary models[c], plus smoking[d]	Primary models[c], plus body mass index[d]
				Adjusted odd ratio per IQR[b] (95% confidence interval)	Adjusted odd ratio per IQR[b] (95% confidence interval)	Adjusted odd ratio per IQR[b] (95% confidence interval)
**UCD_CIT modeled concentrations at the 4km*4km resolution. by fraction and species**						
Nitrates in $PM_{2.5}$	100,794	179,198	2.76	1.172 (1.159, 1.185)	1.172 (1.159, 1.185)	1.170 (1.156, 1.184)
Sulfates in $PM_{2.5}$	100,794	179,198	0.60	1.012 (1.006, 1.019)	1.013 (1.007, 1.019)	1.005 (0.999, 1.011)
**CALINE4 modeled concentrations**						
Ultrafine particle number	100,487	186,905	6355	1.027 (1.018, 1.036)	1.028 (1.019, 1.037)	1.017 (1.008, 1.027)
Carbon monoxide	100,487	186,905	41.99	1.028 (1.018, 1.038)	1.029 (1.019, 1.04)	1.015 (1.004, 1.026)
Nitrogen oxides	100,487	186,905	4.45	1.034 (1.025, 1.043)	1.035 (1.026, 1.044)	1.025 (1.015, 1.034)
**Traffic density (within buffers of different sizes)**						
50m buffer	109,211	210,664		0.947 (0.896, 1.000)	0.948 (0.897, 1.001)	0.907 (0.853, 0.964)
150m buffer	109,211	210,664		0.987 (0.960, 1.015)	0.989 (0.962, 1.017)	0.964 (0.935, 0.993)
250m buffer	109,211	210,664		0.989 (0.963, 1.016)	0.99 (0.965, 1.017)	0.971 (0.943, 0.999)
350m buffer	109,211	210,664		1.000 (0.972, 1.027)	1.001 (0.974, 1.029)	0.982 (0.953, 1.011)
**Distance to roadways**						
Less than 50m	109,225	210,716		0.978 (0.963, 0.993)	0.978 (0.963, 0.993)	0.971 (0.955, 0.988)
Less than 100m	109,225	210,716		0.991 (0.977, 1.004)	0.991 (0.978, 1.004)	0.990 (0.976, 1.004)
Less than 150m	109,225	210,716		0.991 (0.978, 1.005)	0.992 (0.978, 1.005)	0.988 (0.973, 1.002)
Less than 200m	109,225	210,716		0.992 (0.978, 1.007)	0.993 (0.979, 1.007)	0.985 (0.970, 1.000)

a) $PM_{2.5}$; particulate matter less than 2.5 µm in aerodynamic diameter; O_3: ozone; NO_2: nitrogen dioxide; $PM_{0.1}$; particulate matter less than 0.1 µm in aerodynamic diameter; OC: organic carbon; EC: elemental carbon ; SOA secondary organic aerosols.

b) Interquartile range in exposure. Units are micrograms per cubic meter for all particulate mass and elements, part per billion for gaseous pollutants.

c) Odds ratios were estimated using conditional logistic regression models. Primary models were adjusted for race/ethnicity, educational level and for maternal age and median household income at Census block group level using polynomial functions. For estimated pollutant concentrations, odds ratios are expressed per interquartile range. For traffic density, they are expressed per 10,000 vehicles per day per meter. For distance to roadways, they compare births within the stated distance to those outside that distance.

d) Smoking was introduced as a dichotomous variable for "ever smoking during pregnancy" versus "never smoking during pregnancy" whereas body mass index was introduced as a continuous variable.

Table S3. Sensitivity analysis of moderately preterm birth (MPTB, gestational age <35 weeks) or very preterm birth (VPTB, gestational age <30 weeks) and air pollution

	MPTB analysis					VPTB analysis				
Air pollution indicator[a]	Number of cases	Number of controls	IQR[b]	Adjusted odds ratio (95% confidence interval)[c]	p value	Number of cases	Number of controls	IQR[b]	Adjusted odds ratio (95% confidence interval)[c]	p value
**Measured pollutant concentrations interpolated by empirical Bayesian kriging (years 2000-2008)**										
$PM_{2.5}$	151,029	288,868	6.60	1.138 (1.119, 1.156)	< 0.01	27,868	53,309	6.98	1.102 (1.071, 1.134)	< 0.01
Ozone	151,670	291,577	12.22	1.074 (1.060, 1.089)	< 0.01	27,993	53,841	13.99	1.060 (1.034, 1.086)	< 0.01
Nitrogen dioxide	150,909	288,361	10.10	1.077 (1.061, 1.094)	< 0.01	27,878	53,256	10.47	1.048 (1.018, 1.080)	< 0.01
**UCD_CIT modeled concentrations at the 4 km*4 km resolution, by fraction and species (years 2000-2008)**										
Primary $PM_{0.1}$	141,661	254,175	1.420	1.041 (1.030, 1.051)	< 0.01	26,302	47,219	1.477	1.047 (1.024, 1.070)	< 0.01
OC in $PM_{0.1}$	141,661	254,175	1.007	1.037 (1.028, 1.047)	< 0.01	26,302	47,219	1.047	1.044 (1.022, 1.066)	< 0.01
EC in $PM_{0.1}$	141,661	254,175	0.132	1.051 (1.039, 1.063)	< 0.01	26,302	47,219	0.133	1.039 (1.014, 1.065)	< 0.01
SOA in $PM_{0.1}$	141,661	254,175	0.061	1.141 (1.125, 1.158)	< 0.01	26,302	47,219	0.062	1.056 (1.031, 1.082)	< 0.01
Primary $PM_{2.5}$	141,661	254,175	8.308	1.064 (1.053, 1.076)	< 0.01	26,302	47,219	8.582	1.032 (1.007, 1.057)	0.01
OC in $PM_{2.5}$	141,661	254,175	3.766	1.038 (1.027, 1.048)	< 0.01	26,302	47,219	3.929	1.023 (1.000, 1.046)	0.05
EC in $PM_{2.5}$	141,661	254,175	1.262	1.046 (1.035, 1.058)	< 0.01	26,302	47,219	1.280	1.031 (1.006, 1.056)	0.01

Air pollution indicator[a]	MPTB analysis					VPTB analysis				
	Number of cases	Number of controls	IQR[b]	Adjusted odds ratio (95% confidence interval)[c]	p value	Number of cases	Number of controls	IQR[b]	Adjusted odds ratio (95% confidence interval)[c]	p value
SOA in $PM_{2.5}$	141,661	254,175	0.246	1.148 (1.130, 1.166)	< 0.01	26,302	47,219	0.262	1.046 (1.020, 1.074)	< 0.01
Ammonium in $PM_{2.5}$	141,661	254,175	1.194	1.146 (1.130, 1.162)	< 0.01	26,302	47,219	1.219	1.098 (1.069, 1.128)	< 0.01
Nitrates in $PM_{2.5}$	141,661	254,175	2.937	1.142 (1.128, 1.158)	< 0.01	26,302	47,219	3.023	1.092 (1.064, 1.121)	< 0.01
Sulfates in $PM_{2.5}$	141,661	254,175	0.542	1.007 (1.001, 1.013)	0.02	26,302	47,219	0.558	1.005 (0.993, 1.017)	0.45
**UCD_P modeled concentrations at the 4 km*4 km resolution, by species, in $PM_{2.5}$ (years 2000-2006)**										
Potassium	106,821	187,759	0.053	1.044 (1.031, 1.057)	< 0.01	20,058	34,636	0.054	1.034 (1.011, 1.058)	< 0.01
Chromium	106,821	187,759	0.002	1.002 (0.998, 1.006)	0.42	20,058	34,636	0.002	1.005 (0.994, 1.017)	0.36
Iron	106,821	187,759	0.189	1.035 (1.019, 1.052)	< 0.01	20,058	34,636	0.187	1.017 (0.987, 1.048)	0.28
Titanium	106,821	187,759	0.008	1.026 (1.014, 1.038)	< 0.01	20,058	34,636	0.008	1.032 (1.008, 1.057)	0.01
Magnesium	106,821	187,759	0.004	1.021 (1.010, 1.031)	< 0.01	20,058	34,636	0.004	1.029 (1.008, 1.052)	0.01
Strontium	106,821	187,759	0.001	1.019 (1.006, 1.032)	< 0.01	20,058	34,636	0.001	0.989 (0.965, 1.014)	0.38
Arsenic	106,821	187,759	0.001	1.000 (0.998, 1.002)	0.93	20,058	34,636	0.001	0.998 (0.993, 1.003)	0.50
Calcium	106,821	187,759	0.047	1.005 (0.992, 1.019)	0.43	20,058	34,636	0.047	0.993 (0.967, 1.019)	0.58
Zinc	106,821	187,759	0.002	0.997 (0.992, 1.003)	0.39	20,058	34,636	0.002	0.997 (0.980, 1.013)	0.69

Air pollution indicator[a]	MPTB analysis					VPTB analysis				
	Number of cases	Number of controls	IQR[b]	Adjusted odds ratio (95% confidence interval)[c]	p value	Number of cases	Number of controls	IQR[b]	Adjusted odds ratio (95% confidence interval)[c]	p value
**UCD_P modeled concentrations at the 4 km*4 km resolution, by fraction and sources (years 2000-2006)**										
Onroad gasoline $PM_{0.1}$	106,821	187,759	0.083	1.101 (1.080, 1.122)	< 0.01	20,058	34,636	0.083	1.011 (0.975, 1.049)	0.54
Onroad diesel $PM_{0.1}$	106,821	187,759	0.070	1.079 (1.062, 1.097)	< 0.01	20,058	34,636	0.070	1.008 (0.976, 1.041)	0.62
Commercial meat cooking $PM_{0.1}$	106,821	187,759	0.123	1.058 (1.042, 1.074)	< 0.01	20,058	34,636	0.125	1.013 (0.984, 1.043)	0.38
Wood burning $PM_{0.1}$	106,821	187,759	0.274	0.994 (0.984, 1.003)	0.20	20,058	34,636	0.281	0.997 (0.982, 1.012)	0.67
Onroad gasoline $PM_{2.5}$	106,821	187,759	0.386	1.096 (1.076, 1.115)	< 0.01	20,058	34,636	0.385	1.003 (0.970, 1.038)	0.86
Onroad diesel $PM_{2.5}$	106,821	187,759	0.398	1.073 (1.058, 1.089)	< 0.01	20,058	34,636	0.402	1.000 (0.971, 1.029)	0.98
Commercial meat cooking $PM_{2.5}$	106,821	187,759	1.085	1.042 (1.029, 1.055)	< 0.01	20,058	34,636	1.099	0.984 (0.960, 1.009)	0.22
Wood burning $PM_{2.5}$	106,821	187,759	1.830	1.002 (0.991, 1.013)	0.73	20,058	34,636	1.851	0.999 (0.981, 1.016)	0.87
**CALINE4 modeled concentrations (years 2000-2008)**										
In all subjects										
Ultrafine particle number	146,370	273,459	6528	0.994 (0.986, 1.003)	0.17	27,130	50,547	6560	0.988 (0.972, 1.004)	0.14
Carbon monoxide	146,370	273,459	59.22	1.012 (1.002, 1.021)	0.01	27,130	50,547	59.33	1.005 (0.987, 1.023)	0.60
Nitrogen oxides	146,370	273,459	6.00	1.013 (1.005, 1.021)	0.00	27,130	50,547	6.07	1.009 (0.993, 1.026)	0.29

	MPTB analysis					VPTB analysis				
**Air pollution indicator[a]**	**Number of cases**	**Number of controls**	**IQR[b]**	**Adjusted odds ratio (95% confidence interval)[c]**	**p value**	**Number of cases**	**Number of controls**	**IQR[b]**	**Adjusted odds ratio (95% confidence interval)[c]**	**p value**
In subjects geocoded at the tax parcel level										
Ultrafine particle number	78,441	78,944	6806	1.054 (1.041, 1.067)	< 0.01	14,432	14,516	6560	1.039 (1.012, 1.067)	< 0.01
Carbon monoxide	78,441	78,944	65.01	1.082 (1.067, 1.097)	< 0.01	14,432	14,516	59.33	1.065 (1.034, 1.097)	< 0.01
Nitrogen oxides	78,441	78,944	6.52	1.064 (1.051, 1.077)	< 0.01	14,432	14,516	6.07	1.054 (1.026, 1.082)	< 0.01
**Traffic density (within buffers of different sizes) (years 2000-2008)**										
In all subjects										
50m buffer	152,794	296,439		0.942 (0.898, 0.989)	0.02	28,200	54,675		1.096 (0.983, 1.222)	0.10
150m buffer	152,794	296,439		0.982 (0.958, 1.006)	0.14	28,200	54,675		0.996 (0.942, 1.054)	0.90
250m buffer	152,794	296,439		0.967 (0.944, 0.990)	0.01	28,200	54,675		0.944 (0.894, 0.996)	0.04
350m buffer	152,794	296,439		0.956 (0.932, 0.98)	< 0.01	28,200	54,675		0.937 (0.884, 0.993)	0.03
In subjects geocoded at the tax parcel level										
50m buffer	83,375	88,431		1.055 (0.976, 1.140)	0.18	15,253	16,219		1.118 (0.924, 1.352)	0.25
150m buffer	83,375	88,431		1.063 (1.026, 1.101)	< 0.01	15,253	16,219		1.090 (1.000, 1.187)	0.05
250m buffer	83,375	88,431		1.054 (1.018, 1.091)	< 0.01	15,253	16,219		1.039 (0.957, 1.127)	0.36
350m buffer	83,375	88,431		1.064 (1.025, 1.104)	< 0.01	15,253	16,219		1.040 (0.952, 1.136)	0.38

Air pollution indicator[a]	MPTB analysis Number of cases	Number of controls	IQR[b]	Adjusted odds ratio (95% confidence interval)[c]	p value	VPTB analysis Number of cases	Number of controls	IQR[b]	Adjusted odds ratio (95% confidence interval)[c]	p value
**Distance to roadways (years 2000-2008)**										
In all subjects										
Less than 50m	152,838	296,609		0.988 (0.976, 1.001)	0.07	28,204	54,695		0.992 (0.963, 1.022)	0.60
Less than 100m	152,838	296,609		0.998 (0.986, 1.009)	0.70	28,204	54,695		0.989 (0.963, 1.015)	0.40
Less than 150m	152,838	296,609		1.000 (0.988, 1.012)	0.99	28,204	54,695		0.990 (0.964, 1.017)	0.47
Less than 200m	152,838	296,609		0.994 (0.981, 1.006)	0.32	28,204	54,695		0.975 (0.948, 1.003)	0.08
In subjects geocoded at the tax parcel level										
Less than 50m	83,403	88,514		0.990 (0.969, 1.013)	0.39	15,254	16,226		0.970 (0.920, 1.022)	0.26
Less than 100m	83,403	88,514		1.020 (1.002, 1.039)	0.03	15,254	16,226		1.012 (0.970, 1.056)	0.57
Less than 150m	83,403	88,514		1.028 (1.010, 1.046)	< 0.01	15,254	16,226		1.040 (0.998, 1.084)	0.06
Less than 200m	83,403	88,514		1.031 (1.012, 1.051)	< 0.01	15,254	16,226		1.026 (0.983, 1.072)	0.24

a) $PM_{2.5}$; particulate matter less than 2.5 µm in aerodynamic diameter; $PM_{0.1}$; particulate matter less than 0.1 µm in aerodynamic diameter; OC: organic carbon; EC: elemental carbon; SOA secondary organic aerosols

b) Inter-quartile range in exposure. Units are micrograms per cubic meter for all particulate mass and elements, part per billion for gaseous pollutants.

c) Odds ratios were estimated using conditional logistic regression models, adjusted for race/ethnicity, educational level and for maternal age using categorical variables and for median household income at Census block group level using polynomial functions. For estimated pollutant concentrations, odds ratios are expressed per interquartile range. For traffic density, they are expressed per 10,000 vehicles per day per meter. For distance to roadways, they compare births within the stated distance to those outside that distance.

References

Greenland S, Pearl J, Robins JM. 1999. Causal diagrams for epidemiologic research. Epidemiology 10(1): 37-48.

Hu J, Zhang H, Chen SH, Ying Q, Wiedinmyer C, Vandenberghe F, et al. 2014. Identifying PM2.5 and PM0.1 sources for epidemiological studies in California. Environ Sci Technol 48(9): 4980-4990.
